# Myosin1f-mediated neutrophil migration contributes to acute neuroinflammation and brain injury after stroke in mice

**DOI:** 10.1186/s12974-019-1465-9

**Published:** 2019-04-10

**Authors:** Yan Wang, Haojie Jin, Weifang Wang, Feng Wang, Heng Zhao

**Affiliations:** 1 Department of Neurosurgery, Stanford University School of Medicine, 1201 Welch Road, MSLS Building, Room P306, Stanford, CA 94305 USA; 2 Department of Plant Biology, Carnegie Institution for Science, Stanford, CA 94305 USA; 3 Center for Microbiota and Immunological Diseases, Shanghai General Hospital, Shanghai Institute of Immunology, Shanghai Jiao Tong University School of Medicine, 280 South Chongqing Road, Building 5#, Room 807#, Shanghai, 200025 China

**Keywords:** Ischemic stroke, Neuroinflammation, Myosin1f, Neutrophils, Migration

## Abstract

**Background:**

During the acute stroke phase, neutrophils from the peripheral blood are first to arrive in the ischemic brain, which then attracts other immune cells that exacerbate neuroinflammation in the ischemic tissue. Myosin1f was reported to specifically mediate neutrophil migration in the peripheral tissues, but whether it plays a critical role in the neuroinflammatory response after ischemic stroke remains unknown. In this study, we aim to test the hypothesis that myosin1f-mediated neutrophil migration is critical in acute neuroinflammation induced by ischemic stroke.

**Methods:**

Myosin1f ^−/−^ and wild type (WT) mice were subjected to transient middle cerebral artery occlusion (MCAO). To determine which cells determine myosin1f’s transmigration ability, bone marrow transplantation, neutrophil depletion, and adoptive neutrophil transfer were performed. The myosin1f RNA level was assessed in peripheral neutrophils by reverse transcription polymerase chain reaction (RT-PCR) at 1 day and 3 days after stroke. The infiltrating neutrophils were quantified by immunofluorescence staining and FACS at 72 h after reperfusion.

**Results:**

The myosin1f ^−/−^ mice had significantly smaller infarctions than the myosin1f ^+/+^ mice. Bone marrow transplantation from myosin1f ^−/−^ mice to recipient mice also had smaller infarctions compared to animals receiving bone marrow from myosin1f ^+/+^ mice. By performing neutrophil depletion and adoptive transfer, we confirmed that myosin1f acts mainly in circulating neutrophils. RT-PCR showed that myosin1f gene expression was increased in the circulating blood neutrophils at 3 days after ischemia. The confocal immunostaining and FACS results confirmed that fewer neutrophils infiltrated into the ischemic brain in myosin1f ^−/−^ mice compared to WT mice.

**Conclusions:**

Myosin1f determines neutrophil migration into the ischemic hemisphere, which directly affects stroke outcome.

**Electronic supplementary material:**

The online version of this article (10.1186/s12974-019-1465-9) contains supplementary material, which is available to authorized users.

## Introduction

Ischemic stroke is the most common acute cerebrovascular disease worldwide and accounts for approximately 80% of stroke cases [1]. However, the two major FDA-approved therapeutics, the blood clot dissolvent t-PA, and mechanical devices used to remove blood clots, are only available for a small portion of stroke patients with restrictive conditions [2]. Therefore, it is necessary to study leukocyte-mediated neuroinflammatory mechanisms that modulate stroke outcomes to provide new avenues for effective therapeutics against stroke.

After a stroke, the injured neurons release inflammatory cytokines, which recruit immune cells crossing the disrupted brain-blood barrier (BBB) into the ischemic brain [3]. The circulating immune cell migration starts within a few hours of stroke onset, and neutrophils are among the first circulating leukocytes to arrive at the ischemic hemisphere. Previous studies have reported that neutrophil depletion attenuates infarction size in mouse models [4]. In addition, neutrophil migration also plays an essential role in stroke outcome. For instance, neutrophil migration inhibited by blocking the very-late-antigen-4 (VLA-4) reduces brain injury and behavioral impairment [5]. Clinically, diffusion-weighted MR imaging showed that the more neutrophils migrate into the ischemic hemisphere, the higher chance of larger infarct volumes in acute phase after stroke [6].

The class I myosin (myosin1) is a subgroup of the myosin superfamily and is evolutionarily conserved in both mice and human beings [7, 8]. The fibrous myosin proteins consist of three major domains: the head, the converter, and the tail. While the tail domains enable myosin1 to bind to cell membrane lipids, its head domains are able to bind to actin filament, which may connect with chromatin in the nuclei [9]. It contains eight class I myosin members, six of which have short-tailed forms (myosin1a, b, c, d, g, and h), and two of which have long-tailed forms (1e and f). The short-tailed forms are involved in more specialized functions, i.e., vesicles transportation, the adaptation of hair cells in the ear [9, 10], and structure maintenance [11]; while the long-tailed forms function in cell motility and migration [8]. Myosin1f interacts with actin filaments to form an actin-myosin complex within the cytoskeleton of leukocytes to enable the transendothelial and interstitial migration of leukocytes [8]. Myosin1f is selectively expressed in the peripheral immune organs, such as the spleen, lymph nodes, and thymus [8]. Within the lymphoid tissue, only neutrophils selectively express myosin1f, while other leukocytes, such as natural killer (NK) cells, dendritic cells, and macrophages express both forms of long-tailed myosin1f and 1e [8]. A recent report shows that myosin1f is specifically required for neutrophil migration in a 3D environment during the acute inflammation phase of peritonitis and lung injury in the mouse model [12]. Based on these previous studies, we aimed to test the hypothesis that myosin1f-mediated neutrophil migration modulates neuroinflammation and brain injury after stroke.

## Methods

### Animals

Male WT C57BL/6J and myosin1f^−/−^ mice were purchased from The Jackson Laboratory (Bar Harbor, ME), then bred and housed at the Stanford Medical School Animal Care Facility. Experimental protocols were approved by the Stanford University Administrative Panel on Laboratory Animal Care (APLAC). Experiments were performed in accordance with the ARRIVE guidelines and the National Institutes of Health (NIH) Guidelines for the Care and Use of Laboratory Animals with local government approval [13]. A total of 98 male mice were included in this study. All of the animals were randomly assigned to different groups. Animals were excluded according to the following criteria: (1) had no neurological deficits after stroke; (2) brains had evidence of surgical subarachnoid hemorrhage; (3) the bone marrow-transplanted mice did not show CD45.2^+^ by FACS. For all animal studies, the surgeon did not perform the pre-treatments (including bone marrow transplantation, neutrophil depletion, and adoptive transfer) on the mice.

### Focal cerebral ischemia

Anesthesia was induced by 5% isoflurane and maintained at 1–2% isoflurane throughout surgery. Body temperature was maintained at 37 ± 0.5 °C with a surface heating pad during the entire procedure. Focal cerebral ischemia was induced by 45 min transient MCA occlusion by inserting a silicone-coated 6–0 monofilament (Doccol Corp, Redlands, CA) into the left CCA to block the MCA, as we have reported previously [14]. Sham-operated mice underwent the same procedure, but without monofilament insertion. The cerebral blood flow (CBF) was detected before stroke onset, during MCA occlusion, and after reperfusion by using laser Doppler probe. The details are listed in Additional file 1: Table S1.

### Infarction measurement

At 48 h or 72 h after ischemia, the mice were deeply anesthetized with isoflurane and euthanized. The brains were sliced into 5 slices with 2-mm thickness and stained in 2% 2, 3, 5-triphenyl tetrazolium chloride (TTC, ^#^T8877, Sigma Aldrich, St Louis, MO) for 10–15 min at 37 °C, and fixed in 4% PFA overnight. The brain infarctions were then measured using NIH ImageJ software [15]. It was normalized to the contralateral hemisphere and expressed as a ratio according to the following formula: (area of non-ischemic hemisphere − area of non-ischemic tissue in the ischemic hemisphere)/area of non-ischemic hemisphere [16].

### Neurobehavioral examination

Neurobehavioral tests were carried out by an investigator who was blinded to the treatment. This assay is based on a modified neurological severity score (mNSS) system to present a comprehensive neurological function assessment, including motor, sensory, balance, and reflex tests [17]. mNSS scores range from 0 to 14, in which 0 represents normal and 14 represents the highest degree of neurological deficiency. For the motor assay, after raising the mouse by the tail, the bend and torsion of limbs were observed (score 0–3). The walking posture was also checked (score 0–3). For the balance test, mice were placed on a beam to see whether the mouse could keep their balance, if their limbs fell off the beam, and they could walk on the beam (score 0–6). For the sensory and reflex tests, pinna and corneal reflexes were examined, respectively (score 0–2).

### Bone marrow transplant

For the bone marrow chimera experiments, B6.SJL-Ptprca Pepcb/ BoyJ (CD45.2^−^) mice (stock number 002014) were purchased from Jackson Laboratory (Bar Harbor, ME). Further details are found in our previous publication [18]. Briefly, male wild-type B6.SJL mice were subjected to irradiation (950 rad), then 5 × 10^6^ bone marrow cells from wild-type C57BL/6J (CD45.2^+^) or Myosin1f^−/−^ (CD45.2^+^) mice were IV injected into each irradiated recipient. Eight weeks after bone marrow transfer, peripheral blood CD45.2 expressions were detected by flow cytometry to ensure the successful bone marrow transfer.

### Neutrophil depletion

For the neutrophil depletion experiment, In vivo Mab anti-mouse Ly6G (clone 1A8) (Bio X Cell, W. Lebanon, NH) antibody was IP injected 1 day before and 1 day after surgery to induce neutrophil depletion (250 μg/mice). The neutrophil population was then monitored using FACS.

### Neutrophil and monocyte purification

Neutrophils and monocytes were collected from both bone marrow and spleen. Bone marrow cells were flushed from the femora and tibia; the spleen cells were harvested from male C57BL/6J mice (myosin1f ^+/+^) or myosin1f ^−/−^ mice. Cells were then treated with ACK lysis buffer (Invitrogen, Carlsbad, CA) to lyse the red blood cells. After washing with PBS, cells were stained with PE-conjugated Ly6G antibody (clone 1A8) (BioLegend, San Diego, CA) on ice for 20 min. The cells were washed again with PBS, and then labeled with magnetic anti-PE microbeads (Miltenyi Biotec, Sunnyvale, CA) for 15 min at 4 °C. The cells were then separated using a MACS column and MACS separator, according to the manufacturer’s instructions. For monocyte purification, PE-conjugated F4/80 antibody (clone BM8) (BioLegend, CA) was used as the primary antibody, followed by labeling with magnetic anti-PE beads, as indicated above.

### Neutrophil adoptive transfer

B6.SJL-Ptprca Pepcb/BoyJ (CD45.2^−^) mice were used as the recipient mice for the adoptive neutrophil transfer experiments. Neutrophils were first depleted using anti-mouse Ly6G (clone 1A8) via IP injection (250μg/mice). After 36 h, purified neutrophils from myosin1f^−/−^ mice (CD45.2^+^) or wild-type C57BL/6J mice (myosin1f ^+/+^, CD45.2^+^) were adoptively transferred via IV injection. The CD45.2 expression was monitored by fluorescence-activated cell sorting (FACS).

### Real-time PCR

To evaluate the myosin1f expression levels after stroke, neutrophils were first collected from whole blood in the sham control group 1 day and 3 days after stroke. Whole blood was collected into pretreated K_2_ EDTA tubes (BD Life Sciences, Franklin Lakes, NJ). The red blood cells were lysed with ACK lysis buffer and then selected using MACS magnetic beads (details in neutrophils purification). RNA was purified using an RNeasy Mini Kit (Qiagen, Valencia, CA). The RNA quality was measured using the Bioanalyzer. The relative expression of myosin1f were performed using the TaqMan method at the Stanford Pan facility. Myosin1f TaqMan primer (Assay ID: Mm01271176_m1) and reference gene β-actin (Assay ID: Mm00615581_s1) were purchased from Thermo Fisher Scientific (Waltham, MA). The relative ICAM-1 and MAC-1 expressions were measured using SYBR green products-based RT-PCR. The primers were synthesized from the Stanford Pan facility. The detailed primer sequences are listed in Additional file 1: Table S2.

### Neutrophils transmigration assay

Neutrophils were first purified from myosin1f^−/−^ mice or wild-type C57BL/6J mice bone marrow using MACS magnetic beads (details in neutrophils purification). Neutrophil transmigration was analyzed using transwell plates (Corning, Tewksbury, MA). Twenty-micrometer N-formyl-methionyl-leucyl-phenylalanine (fMLP) was added in the lower chamber, and cells were treated using fibronectin protein, as previously described [8, 12]. After 3 h incubation by 5% CO2 at 37 °C, the cells that migrated into the lower chamber were counted. Briefly, 100 μL of trypan blue-treated cell suspension was applied to a glass hemocytometer. Using a microscope, the hemocytometer gridlines were viewed with a × 10 objective and the cells were counted.

### Immunostaining

After a stroke, mice were transcardially perfused with PBS and fixed in 4% (*w*/*v*) paraformaldehyde. Mouse brains were cut into 30 μM coronal slices using a microtome (Leica3050, Wetzlar, Germany). For staining, brain slices were washed in 0.3% Triton for 5 min and blocked with BSA at room temperature for 2 h. After removing the blocking buffer, a 1:200 dilution of primary antibody, anti-myeloperoxidase antibody (MPO) (Abcam, Cambridge, MA) was added and incubated overnight at 4 °C. To remove the primary antibody, the slices were washed three times for 15 min with PBS. A 1:200 dilution of Alexa 488-conjugated goat anti-mouse antibody (Invitrogen, Carlsbad, CA) was added and then incubated at room temperature for 2 h in the dark. The secondary antibody was removed by washing the slices three times in PBS, mounted with DAPI (Vector Laboratories, Burlingame, CA), and stored with a coverslip. Fluorescent images were taken by a confocal microscope (Zeiss LSM510, Jena, Germany) and analyzed using Zen software (https://www.zeiss.com/microscopy/int/products/microscope-software/zen-lite.html).

#### Fluorescence-activated cell sorting

The brain immune cells were isolated, as described previously [19]. In brief, mice were deeply anesthetized with isoflurane, euthanized, and then transcardially perfused with cold PBS. The ischemic hemispheres were collected for FACS staining. For sham control, the whole brain was collected. The brain tissues were mildly homogenized on ice and centrifuged at 1800 rpm for 5 min. Thirty percent Percoll was then added, centrifuged twice for 4 min, and then washed with PBS. For FACS intercellular staining, the cells rested for 2 h at 37 °C in 5% CO2, then washed with PBS. The cells were then stained with live/dead aqua (Thermo Fisher Scientific, Waltham, MA) on ice in the dark for 10 min, and then stained on ice for 30 min with FITC anti-mouse CD45 (clone 30-F11), Pacific blue anti-mouse/human CD11b (clone M1/70), and PE anti-mouse Ly6G (clone 1A8). Cells were fixed and permeabilized using intracellular flow cytometry (all the antibodies and fix buffer were purchased from BioLegend).

### Statistics

Animal numbers were calculated prior to the experiments using G*Power software [20]. The comparison of the difference between two independent means with two tails was analyzed, using a priori with given α (0.05) and power (0.95). The estimated average mean for wild type was set to be 39 (with SD ± 5) and the difference above 25% (mean value for knock out ~ 27 with SD ± 5) was considered as a significant difference. The estimated number of samples for each group is five or more. All results are presented as mean ± SD. Statistical significance of differences among groups was determined by two-tailed unpaired and paired Student’s *t* test. For experiments with one treatment and more than three groups, one-way ANOVA was applied. For experiments with more than one treatment or variants, two-way ANOVA was performed followed by Tukey’s multiple comparisons test using GraphPad Prism 7.0 (GraphPad Software, La Jolla, CA. www.graphpad.com).

## Results

**Fig. 1:**
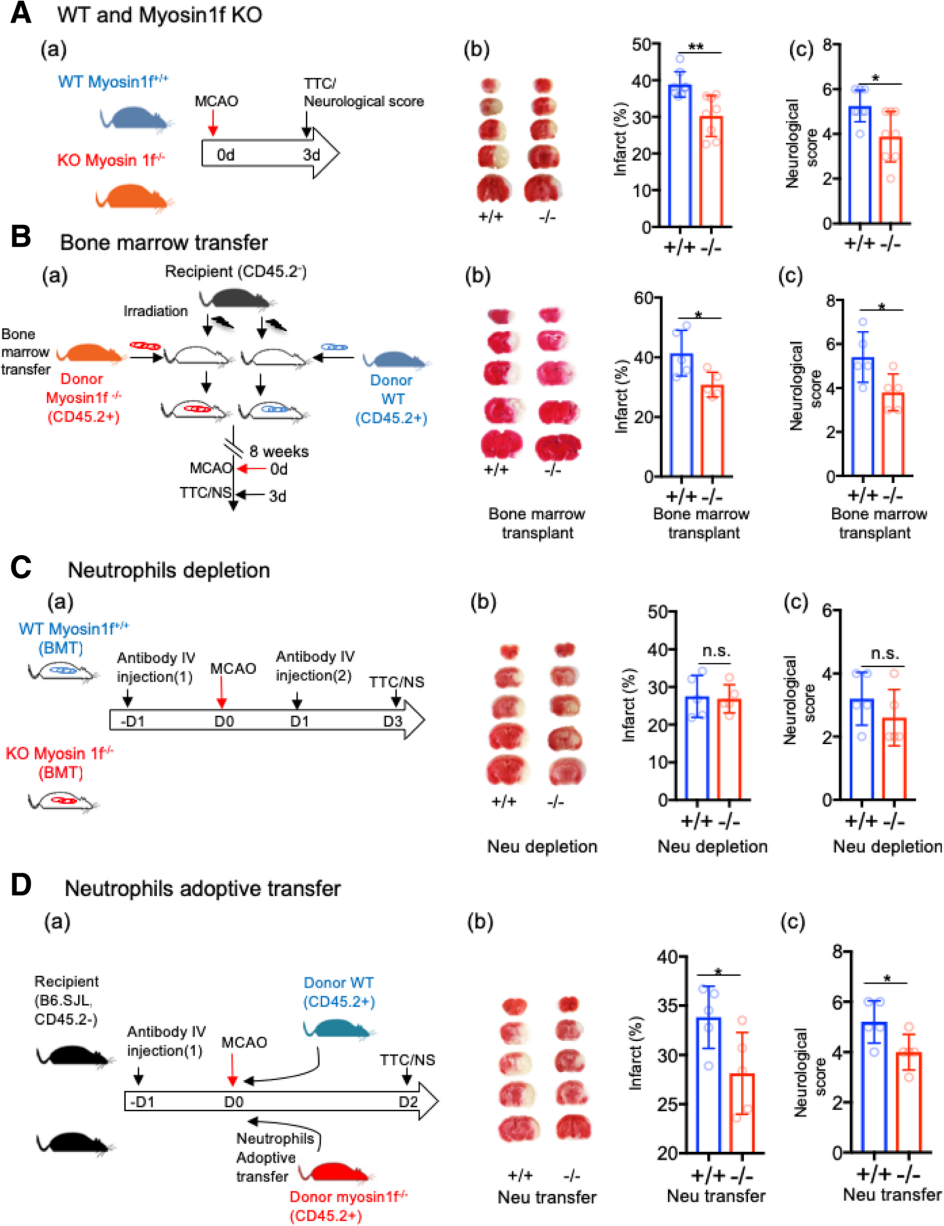
Myosin1f affects ischemic brain injury through circulating neutrophils. **A** Myosin1f KO mice has smaller infarction. (a) The experimental procedure. The WT mice are indicated in blue; myosin1f KO mice are indicated in red. (b) Representative TTC-stained brain infarction and quantification of infarct size, which are expressed as percentages to the ischemic hemisphere (*p* = 0.023). (c) Neurological scores measured 3d after stroke (*p* = 0.0111, *n* = 8 mice/group). **B** Circulating leukocytes are critical for the protective effects of myosin1f^−/−^ against stroke. (a) Experimental procedures to study the effect of bone marrow transfer on stroke outcomes: after irradiation, recipient WT mice (CD45.2^−^, myosin1f^+/+^) received bone marrow cells from WT mice (CD45.2^+^, myosin1f^+/+^) or myosin1f^−/−^ mice (CD45.2^+^, myosin1f^−/−)^, respectively. Eight weeks later, animals were subjected to MCAO; the infarction and neurological scores were assessed at 3 days after stroke. (b) Infarct sizes: representative TTC staining and quantification of infarctions (*p* = 0.0263). (c) Neurological score. (*p* = 0.0353, *n* = 5 mice/group). **C** The effects of circulating neutrophil depletion. (a) Experimental procedures: Ly6G antibodies were IV injected 1 day before and 1 day after stroke to deplete neutrophils. (b) Infarct sizes. *N* = 5 mice/group. (c) Neurological scores. **D** Adoptive transfer of myosin1f^−/−^ neutrophils attenuated brain infarction. (a) Experimental procedures to study the effects of neutrophil transfer on stroke outcomes: the recipient mice were IV injected with Ly6G antibodies for neutrophil depletion. The animals then received adoptive transfer of purified neutrophils from WT and myosin1f^−/−^ mice. Animals were euthanized 2 days later. (b) Infarct sizes (*p* = 0.0409, *n* = 5 mice/group). (c) Neurological scores (*p* = 0.0400, *n* = 5 mice/group)

Our data showed that myosin1f ^−/−^ mice had significantly smaller infarctions compared to WT mice (Fig. 1A). As the previous study showed that myosin1f is mainly expressed in the peripheral lymphoid organ but not in the brain [8], suggesting that myosin1f is mainly involved in immune cells and tissues, and it is very likely that myosin1f affects brain injury via circulating leukocytes. To test this, bone marrow chimeric mice were constructed (Fig. 1B), and the results show that the recipient mice receiving bone marrow cells from myosin1f ^−/−^ mice had less brain damage compared to animals receiving myosin1f ^+/+^ bone marrow cells, suggesting that circulating leukocytes are critical for myosin1f in determining stroke outcomes.

To identify whether myosin1f in neutrophils directly affects stroke outcomes, we conducted a two-step experiment. First, neutrophils were depleted to test the role of neutrophils in stroke outcomes. Ly6G antibodies were IP injected into bone marrow-transplanted mice for two consecutive days, at 1 day before and 1 day after stroke to ensure the successful depletion of Ly6G^+^ cells (Fig. 1C; Additional file 1: Figure S1). The results show that after neutrophil depletion, the infarction sizes were both decreased and comparable between myosin1^−/−^ and myosin1f ^+/+^ mice. Second, to further confirm the role of myosin1f in neutrophils, the purified neutrophils from myosin1f ^+/+^ and myosin1f ^−/−^ mice (CD45.2^+^) were adoptively transferred to recipient mice (CD45.2^−^), respectively, whose neutrophils had been depleted by the injection of Ly6G antibodies (Fig. 1D). The infarctions were measured 2 days after the adoptive transfer based on pre-measured time courses (Additional file 1: Figure S2). Our results showed that adoptive myosin1f ^−/−^ neutrophil transfer resulted in smaller infarction compared with myosin1f ^+/+^ neutrophil transfer in mice (Fig. 1D).

**Fig. 2:**
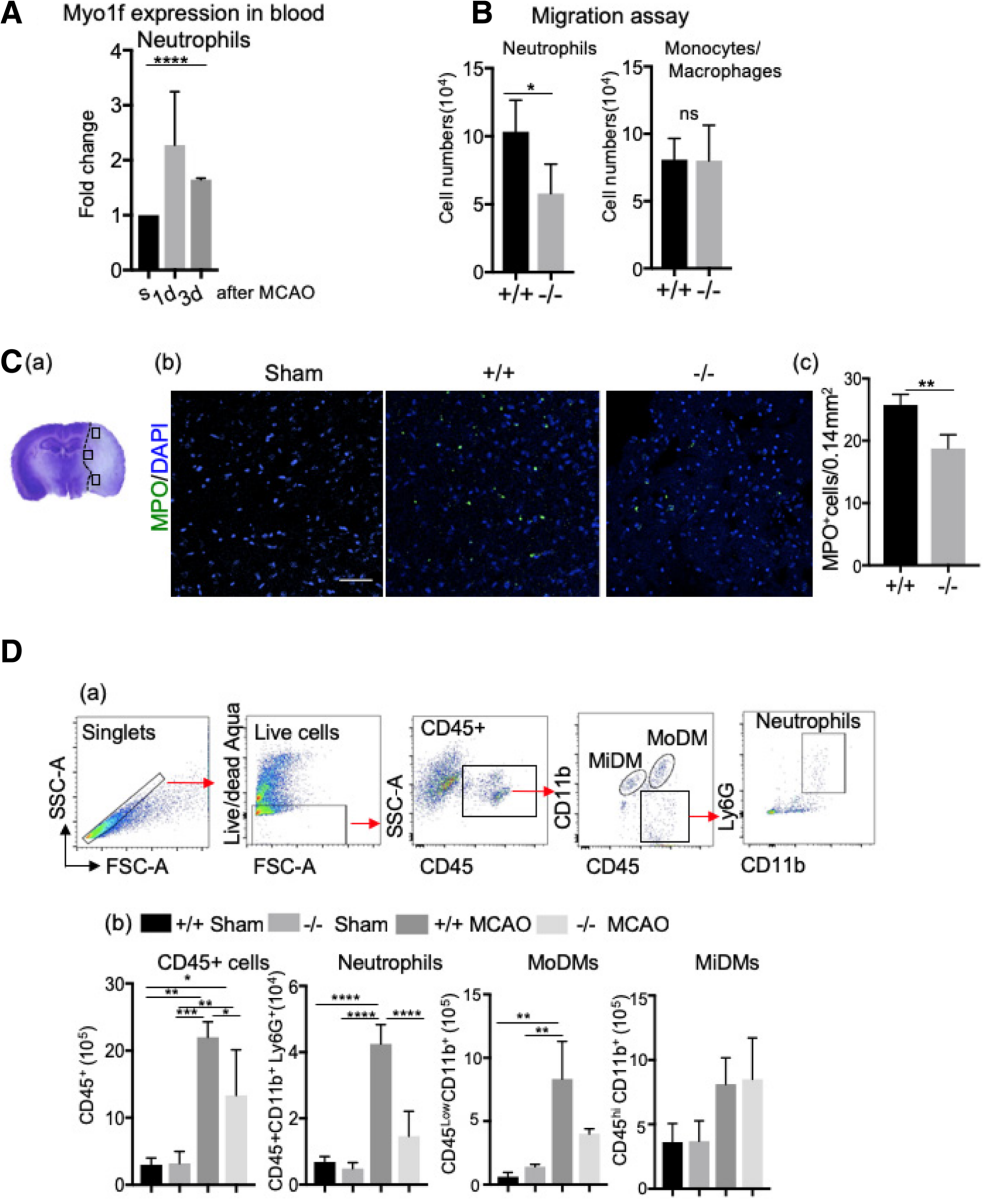
Myosin1f is required for neutrophil migration after acute ischemic stroke. **A** Myosin1f gene expression increased in blood neutrophils at 3 days after stroke compared to the sham control group(s), **** *p* < 0.0001, *n* = 3 mice/group; each has 3 replicates. **B** In vitro migration assay: the number of myosin1f ^+/+^ neutrophils migrated from the upper chamber to the lower chamber was significantly higher compared to myosin1f^−/−^ neutrophils. *p = 0.0463, *n* = 3–4 mice/group; each has 3 replicates. **C** Confocal immunofluorescent staining for MPO, which was counterstained with DAPI. (a) A representative brain section stained with cresol/violet is presented to show the three indicated regions where cell numbers were quantified. (b) Representative staining of MPO^+^ (green) and DAPI (blue) at 72 h after stroke. (c) The bar graph represents the statistic results of the MPO^+^ positive cells. Scale bar: 50 μm. ** *p* = 0.0024, *n* = 5–6 mice/group. **D** FACS quantification of infiltrating neutrophils in the ischemic brain. (a) The representative gating strategies of CD45^+^, MiDMs (microglia-derived macrophages, CD45^Low^CD11b^+^), MoDM (monocytes-derived macrophages, CD45^Hi^CD11b^+^), and neutrophils (CD11b^+^Ly6G^+^). (b) The cell numbers of total CD45^+^ leukocytes, neutrophils, MiDMs, and MoDMs. (*, **, ***, ****, *p* < 0.05, 0.01, 0.001, 0.0001, respectively. *N* = 3–5 mice/group.)

Indeed, the RT-PCR analyses showed the peripheral circulating neutrophil myosin1f expression was increased at 3 days after ischemic stroke compared to sham control (Fig. 2A). In addition, in vitro migration assay showed that purified neutrophils from myosin1f ^−/−^ mice had decreased migration ability compared to WT mice, but not in monocytes (Fig. 2B). This confirms that myosin1f is necessary for peripheral neutrophil migration but not monocytes after ischemia. The immunofluorescence staining (IF) showed that stroke resulted in a significant decrease of MPO^+^ cells in myosin1f ^−/−^ mice (Fig. 2C). The FACS results further confirmed that both total immune cell numbers and infiltrating neutrophil numbers decreased in myosin1f^−/−^ mice. The number of monocyte-derived macrophages (MoDMs) also showed a reduction (Fig. 2D).

It is known that the intracellular adhesion molecule 1 (ICAM-1) and macrophage-1antigen (MAC-1) are functionally required for neutrophils and other leukocytes during adhesion and crawling [21–23], we therefore further assessed their expression levels. We used RT-PCR to measure these gene expressions in purified neutrophils at 3 days after stroke. The results show that both ICAM-1 and MAC-1 did not show a significant difference (Additional file 1: Figure S3), demonstrating that neutrophil extravasation was not dependent on the expression level changes of these factors, but more likely related to myosin1f.

## Discussion

In our current study, we showed that myosin1f KO specifically attenuates neutrophil migration ability, both in vitro and in vivo. Myosin1f ^−/−^ mice had smaller infarction compared to WT mice. Moreover, recipient mice receiving myosin1f ^−/−^ neutrophils had better stroke outcome compared to animals receiving myosin1f ^+/+^ neutrophils.

Stroke-induced ischemic injury triggers a robust inflammatory response in a few hours after blood vessel occlusion. This inflammation starts with a local reaction that includes inflammatory cytokines and chemokines released from injured neurons and brain-resident microglia, followed by the recruitment of leukocytes from peripheral circulation [24, 25]. Neutrophils are rapidly mobilized from bone marrow after stroke and are the first cell type to arrive in the ischemic brain, thereby initiating an efficient innate immune response that peaks around 2 days to 3 days [26, 27]. Neutrophil infiltration is associated with increased infarction size, brain-blood barrier (BBB) disruption, and reduced neurological scores in both mice and patients [6, 28, 29]. Previous studies showed that direct neutrophil depletion using antibodies reduces the BBB breakdown, attenuates brain injury, and decreases brain inflammation in both intracerebral hemorrhage and focal cerebral ischemia mice models [4, 30].

Other experimental evidence shows that disrupting neutrophil infiltration also results in significant protection after stroke. There are three main steps for neutrophil infiltration including capture and rolling, adhesion and crawling, and transmigration [31]. The first two steps have been well studied [32]. For instance, blocking the very-late-antigen-4 (VLA-4) effectively inhibits neutrophil interaction with endothelial cells, and significantly decreases brain injury [5]. In addition, the neutrophil adherence antagonist, WEB2086, which inhibits the platelet-activating factor (PAF) and blocks the leukocyte adhesion to endothelial cells, showed beneficial effects during spinal cord ischemia-reperfusion in rabbits [33]. Moreover, the two molecules, intracellular adhesion molecule 1 (ICAM-1), and macrophage-1antigen (MAC-1) are functionally required for neutrophils and other leukocytes during adhesion and crawling [32]. The ICAM-1 levels are increased after stroke in both animals and patients [21, 22], and blocking ICAM-1using antibodies reduces ischemic damages after transient MCAO in rats [23]. Genetic depletion of ICAM-1 also decreases infarct size and improves outcomes in a rodent model [34]. MAC-1 blocks by using neutrophil inhibitory factor (NIF) results in smaller infarction and functional outcome improvement in rats [35, 36]. Nevertheless, the molecular mechanisms of neutrophil transmigration remain less studied compared with the first two stages of leukocyte recruitment [32].Thus, we investigated the role of myosin1f in neutrophils transmigration.

A recent study showed that myosin1f is specifically required for neutrophil migration in acute peritonitis and acute lung injury mouse model [12]. In line with this study, our data confirmed that myosin1f KO attenuates neutrophil migration into the ischemic hemisphere. The downregulation of migration ability was only found in neutrophils rather than in monocytes. One possible reason is that myosin1f is the only long-tailed isoform expressed in neutrophils, while both 1e and 1f are expressed in other cells type, including monocytes/macrophages, NK cells, and dendritic cells [8]. The two isoforms, myosin 1e and 1 f, are not functionally identical. Myosin1f was reported to specifically regulate neutrophil migration, but did not affect neutrophil phagocytosis [8]. For instance, myosin1e is involved in phagocytosis and phagosome closure [37, 38], but myosin1f is not [8]. In addition, myosin1e is required for plasma membrane tension, but myosin1f is not required for cortical tension, one of the main characters of plasma membrane tension generation [8, 39]. Compared with myosin1e, myosin1f is specifically essential for neutrophil migration. Our in vivo studies indeed showed that myosin1f ^−/−^ mice had less neutrophils migrating into the ischemic hemisphere after stroke. Using adoptive transfer techniques, we further confirmed that myosin1f in circulating neutrophils plays the key role, given that there is no myosin1f expression in the brain [8].

Our RT-PCR results showed that the myosin1f expression was significantly increased in peripheral neutrophils during the acute phase of ischemic stroke, indicating that the peripheral neutrophils may require a higher expression level of myosin1f to migrate into the ischemic area. Previous studies have shown that myosin1f is required for neutrophil migration in a 3D environment [12], as it regulates the deformation of the neutrophil nucleus to facilize the migration through physical barriers [12]. Therefore, the increases in myosin 1f expression in peripheral neutrophils are a response corresponding to the requirement of neutrophil migration into the ischemic brain.

Since the myosin1f KO in mice is a global knockout, we cannot exclude the possibility that the neutrophil migration was also influenced in the immune tissues, such as the spleen. Splenectomy has been reported to attenuate brain injury [40]. It served as a major reservoir for circulating immune cells, thus it is a crucial peripheral immune organ that mediates inflammation. Therefore, it is possible that myosin1f gene KO results in fewer neutrophils emigrating from the spleen into the peripheral blood, leading to attenuated infiltration of neutrophils in the ischemic brain. In addition, neutrophils are known to mediate edema formation [41], and edema is a crucial step for brain injury after stroke [42]. We speculate that myosin1f KO may protect against brain injury by attenuating brain edema by inhibiting neutrophil infiltration.

We are aware that our current study has some limitations. For instance, only young male adult animals were used, but gender has a significant influence on the stroke outcomes [43]. In addition, aging has a great impact on neuroinflammation [44]. Therefore, future experiments should be conducted by including female and aged animals.

## Conclusion

We showed that the myosin1f^−/−^ mice had significantly smaller infarctions than the myosin 1f^+/+^ mice, that peripheral neutrophils are the key cell types responsible for the effects of myosin1f on brain injury, and that myosin1f determines neutrophil migration into the ischemic hemisphere. We therefore conclude that myosin1f is an important factor for determining neutrophil migration into the ischemic brain, and it is a potential target for attenuating brain injury induced by stroke.

## Additional file

Additional file 1: F**igure S1.** Representative flow cytometry analysis of neutrophil depletion. Single cells were obtained by gating with FSC-height vs. FSC-area, and then live/dead cells were gated by using Aqua to exclude dead cells. Peripheral blood neutrophils were first gated using FSC-A and SSC-A (for granulocytes) and then confirmed as CD45^+^ and Ly6G^+^ cells. Figure S2. A. Experimental procedures. For neutrophil depletion, the recipient mice (CD45.2-) were IP injected with Ly6G antibodies on 1 day. The animals then received an adoptive transfer of purified neutrophils from WT type (CD45.2+) on 0 day. Then FACS analysis of the CD45.2 cells in the peripheral blood 36 h and 72 h after adoptive neutrophil transfer. B. 36 h after transfer, a group of cells still expressed CD45.2 but then stopped after 72h. Figure S3. The comparison of relative gene expression for genes ICMA-1 (A) and MAC-1 (B) in blood neutrophils at 3 days after stroke between myosin1f ^+/+^ (+/+) and myosin1f^−/−^ (−/−) mice. *n* = 3/group; each has 3 replicates. There was no significant difference. Table S1. Cerebral blood flow (CBF) was measured before stroke onset, during stroke, and after reperfusion. Table S2. Primer sequences. (DOC 338 kb)

## Abbreviations

APLAC: University Administrative Panel on Laboratory Animal Care
BBB: Brain-blood barrier
CCA: Common carotid artery
FACS: Fluorescence-activated cell sorting
ICAM-1: Intracellular adhesion molecule 1
IF: Immunofluorescence staining
MAC-1: Macrophage-1antigen
MCAO: Middle cerebral artery occlusion
mNSS: A modified neurological severity score
MPO: Myeloperoxidase antibody
NIF: Neutrophil inhibitory factor
NK: Natural killer cells
PAF: Platelet-activating factor
RT-PCR: Reverse transcription polymerase chain reaction
VLA-4: Very-late-antigen-4
WT: Wild type
