# Supplementary material for: Myosin1f-mediated neutrophil migration contributes to acute neuroinflammation and brain injury after stroke in mice
Source: J Neuroinflammation. 2019 Apr 10;16:77. doi: 10.1186/s12974-019-1465-9 (PMC6456973; doi:10.1186/s12974-019-1465-9)
Supplement: Supplementary file 1 — Figure S1. Representative flow cytometry analysis of neutrophil depletion. Single cells were obtained by gating with FSC-height vs. FSC-area, and then live/dead cells were gated by using Aqua to exclude dead cells. Peripheral blood neutrophils were first gated using FSC-A and SSC-A (for granulocytes) and then confirmed as CD45+ and Ly6G+ cells. Figure S2. A. Experimental procedures. For neutrophil depletion, the recipient mice (CD45.2-) were IP injected with Ly6G antibodies on 1 day. The animals then received an adoptive transfer of purified neutrophils from WT type (CD45.2+) on 0 day. Then FACS analysis of the CD45.2 cells in the peripheral blood 36 h and 72 h after adoptive neutrophil transfer. B. 36 h after transfer, a group of cells still expressed CD45.2 but then stopped after 72h. Figure S3. The comparison of relative gene expression for genes ICMA-1 (A) and MAC-1 (B) in blood neutrophils at 3 days after stroke between myosin1f +/+ (+/+) and myosin1f−/− (−/−) mice. n = 3/group; each has 3 replicates. There was no significant difference. Table S1. Cerebral blood flow (CBF) was measured before stroke onset, during stroke, and after reperfusion. Table S2. Primer sequences. (DOC 338 kb) [file 12974_2019_1465_MOESM1_ESM.doc]

**SUPPLEMENTAL MATERIAL**

**Figure S1**

Before neutrophil depletion

After neutrophil depletion


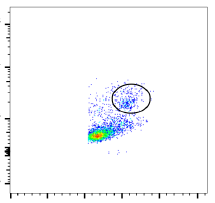

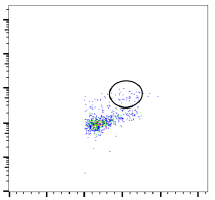


FSC-A

SSC-A

SSC-A

Granulocytes

Granulocytes

CD45

Ly6G


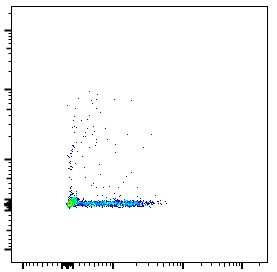

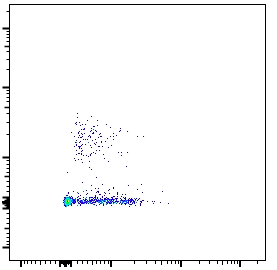


Ly6G

Ly6G+ cells

Ly6G+ cells

**Fig. S1.** Representative flow cytometry analysis of neutrophil depletion. Single cells were obtained by gating with FSC-height vs. FSC-area, and then live/dead cells were gated by using Aqua to exclude dead cells. Peripheral blood neutrophils were first gated using FSC-A and SSC-A (for granulocytes) and then confirmed as CD45^+^ and Ly6G^+^ cells.

Figure S2

Recipient

(CD45.2-)


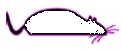


IP

injected ly6G antibody

-1d


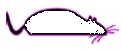

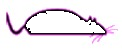


Donor

(CD45.2+)

Neutrophils depletion


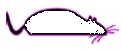


Neutrophils adoptive transfer


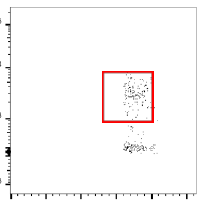

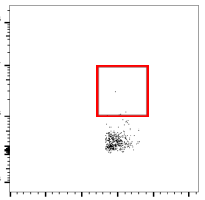


FSC-A

CD45.2

FACS analyses for CD45.2**+** neutrophils

36h 72h

FACS analyses

72h

36h

A

B

0d

**Fig. S2. A.** Experimental procedures. For neutrophil depletion, the recipient mice (CD45.2-) were IP injected with Ly6G antibodies on -1d. The animals then received an adoptive transfer of purified neutrophils from WT type (CD45.2+) on 0d. Then FACS analysis of the CD45.2 cells in the peripheral blood 36h and 72h after adoptive neutrophil transfer. **B.** 36h after transfer, a group of cells still expressed CD45.2 but then stopped after 72h.

Figure S3

A

B

n.s.

n.s.

**Fig. S3.** The comparation of relative gene expression for genes ICMA-1 **(A)** and MAC-1 **(B)** in blood neutrophils at 3d after stroke between myosin1f ^+/+^ (+/+) and myosin1f^-/-^ (-/-) mice. n=3 /group; each has 3 replicates. There was no significant difference.

Table S1

We measured changes in CBF before stroke, during stroke and after reperfusion using a laser Doppler probe. CBF was reduced to ~ 15% at the measured cortex compared to before MCAO. The values of CBF were not significantly different in WT, Myosin1f KO, bone marrow transfer and neutrophils adoptive transfer mice.

Table : Cerebral Blood Flow (CBF) was measured before stroke onset, during stroke, and after reperfusion.

| **Mice** | **Before MCAO** | **During MCAO** | **After MCAO** |
| --- | --- | --- | --- |
| WT | 1.000 ± 0.023 | 0.123 ± 0.023 | 0.932 ± 0.035 |
| Myosin 1f ^-/-^ | 1.032 ± 0.045 | 0.139 ± 0.039 | 0.871 ± 0.046 |
| BMT +/+ | 1.013 ± 0.053 | 0.162 ± 0.023 | 0.843 ± 0.037 |
| BMT -/- | 1.021 ± 0.041 | 0.143 ± 0.031 | 0.874 ± 0.043 |
| Neu depletion +/+ | 0.984 ± 0.032 | 0.157 ± 0.013 | 0.879 ± 0.053 |
| Neu depletion -/- | 0.976 ± 0.041 | 0.146 ± 0.025 | 0.803 ± 0.041 |
| Neu transfer +/+ | 1.043 ± 0.032 | 0.136 ± 0.038 | 0.901 ± 0.027 |
| Neu transfer -/- | 1.031 ± 0.040 | 0.142 ± 0.031 | 0.895 ± 0.021 |

BMT = bone marrow transplant; Neu depletion = neutrophils depletion; Neu transfer =

neutrophil transfer

Table S2: Primer sequences

| Gene | | Sequence |
| --- | --- | --- |
| ICAM-1 | Forward | 5’ GTGGCGGGAAAGTTCCTG 3’ |
|  | Reverse | 5’ CGTCTTGCAGGTCATCTTAGGA 3’ |
| MAC-1 | Forward | 5’ GGATCATAGGCGCCCACTT 3’ |
|  | Reverse | 5’ TCCTTACCCCCACTCAGAGACT 3’ |
